# Treatment outcome of supraglottoplasty vs. wait-and-see policy in patients with laryngomalacia

**DOI:** 10.1007/s00405-016-3943-3

**Published:** 2016-02-29

**Authors:** Martijn van der Heijden, Frederik G. Dikkers, Gyorgy B. Halmos

**Affiliations:** Department of Otorhinolaryngology/Head and Neck Surgery, University of Groningen, University Medical Center Groningen, P.O. Box 30.001, 9700 RB Groningen, The Netherlands; Department of Head and Neck Oncology and Surgery, The Netherlands Cancer Institute-Antoni van Leeuwenhoek, Plesmanlaan 121, 1066 CX Amsterdam, The Netherlands

**Keywords:** Laryngomalacia, Supraglottoplasty, Synchronous airway lesions, Wait-and-see

## Abstract

In most cases, laryngomalacia presents as a mild disease, and the symptoms resolve after wait-and-see policy. Up to 20 % of patients present with severe laryngomalacia and may require surgery (i.e. supraglottoplasty); however, the indication for surgery is not firmly established yet. The goal of this study is to determine whether supraglottoplasty results in a better outcome than wait-and-see and to investigate how different comorbidities influence outcome. A retrospective study of pediatric cases of in a tertiary referral center was performed. Photo and video documentation was available and revised in all cases. Electronic and paper charts were reviewed for the following variables: gender, sex, gestational age, birth weight, symptoms, comorbidity, date of endoscopy, severity and type of laryngomalacia, treatment modality and technique and follow-up data and a total 89 patients were included. Supraglottoplasty was found to lead to significantly faster complete improvement of laryngomalacia than wait-and-see policy (5 weeks vs. 29, *p* = 0.026). Synchronous airway lesions (SALs) were present in 40.4 % of patients and were associated with prolonged symptoms of laryngomalacia (38.5 weeks vs. 14.5, *p* = 0.043). Supraglottoplasty is safe and effective in treatment of severe laryngomalacia. SALs and comorbidities are frequently found in patients with laryngomalacia and are responsible for longer onset of complaints.

**Electronic supplementary material:**

The online version of this article (doi:10.1007/s00405-016-3943-3) contains supplementary material, which is available to authorized users.

## Introduction

Congenital laryngomalacia is the most common etiology causing stridor in infants with a reported incidence of 35–75 % of patients [1]. Supraglottic structures collapse into the airway during inspiration, causing airway obstruction. In most cases laryngomalacia presents with inspiratory, sometimes with in- and expiratory, stridor. The stridor typically worsens with feeding, crying, supine positioning, agitation and exercise. Typically, symptoms are not present at birth, start within the first 4 months of life and peak at an age of 4–8 months [2]. The most common associated symptoms are swallowing dysfunction, regurgitation, cough, sleep-disordered breathing [1, 3]. An estimated 40 % of infants presenting with laryngomalacia have mild and 40 % have moderate symptoms and can be treated conservatively, spontaneously resolving at the age of 12–24 months [1]. Up to 20 % of laryngomalacia patients present with severe laryngomalacia. Severe laryngomalacia is not likely to cure spontaneously, and is more likely to require supraglottoplasty [4].

Laryngomalacia is a self-limiting disease. Nowadays, treatment consists of either wait-and-see or endoscopic surgery (supraglottoplasty). Previous studies have shown that endoscopic surgical treatment is of additional value in the treatment of laryngomalacia [5, 6]. The experiences with complications after supraglottoplasty are controversial in the current literature. Studies related failure and complications directly to comorbidities [7, 8], while other studies have shown otherwise [5, 9, 10]. Various supraglottic structures can be affected in cases with laryngomalacia, as described in the Groningen Laryngomalcia Classification System [11]. Most common findings are: inward collapse of arytenoids cartilages (type 1), medial displacement of aryepiglottic folds (type 2) and posterocaudal displacement of epiglottis against the posterior pharyngeal wall (type 3). Demonstrative video material of these three types are available in the online version of the manuscript (Videos 1–3). Most patients show combinations of these dynamic abnormalities. The type of laryngomalacia determinates the type of supraglottoplasty; type 1 laryngomalacia necessitate removing redundant supra-arytenoid tissue, type 2 incision of the shortened aryepiglottic folds, and type 3 epiglottopexy [11]. These surgical interventions can be combined.

The primary purpose of the present study is to evaluate the best treatment policy in the treatment of laryngomalacia by comparing the short and long-term outcome of patients with moderate and severe laryngomalacia treated by either supraglottoplasty or wait-and-see policy. The secondary aim was to identify predictors of treatment outcome.

## Materials and methods

All patients diagnosed with laryngomalacia between January 2002 and December 2012, less than 5 years of age at the time of diagnosis, were included. All patients were treated at the Department of Otorhinolaryngology, Head and Neck Surgery of the University Medical Center Groningen, The Netherlands, a tertiary referral center. Patients were excluded if they underwent previous surgery for other airway problems; if there was insufficient amount of data for analysis; or when reviewing the endoscopic documentation revealed another diagnosis than laryngomalacia. Imaging material was reviewed by the first author (MvdH) under supervision of the two senior authors (FGD and GBH).

A retrospective chart view of patients with laryngomalacia was performed. Electronic patients’ charts were primarily reviewed, and paper charts were reviewed if additional data were needed. The following variables were collected: gender, gestational age, birth weight, age at diagnosis, presenting symptoms, severity of disease, type of laryngomalacia (according to GLCS [11]), age at endoscopy, synchronous airway lesions, performed treatment, postoperative outcome, time from treatment until free of symptoms, comorbidity and genetic disorders.

When laryngomalacia was suspected, diagnosis was confirmed by flexible fiberoptic laryngoscopy under general anesthesia. Patients were breathing spontaneously throughout the procedure and evaluation of the airway was performed, including assessment of synchronous airway lesions. Rigid endoscopy was not performed routinely, only on indication (e.g. therapeutic procedure). A division of severity was made on clinical presentation and endoscopic findings [7]. Severe laryngomalacia was established if complaints included severe feeding difficulties, failure to thrive, cyanosis or intercostal and/or xyphoidal retractions or endoscopy showed significant dynamic airway obstruction.

Reasons for supraglottoplasty were severe clinical presentation or insufficient spontaneous resolving of symptoms during exerting a wait-and-see policy. Supraglottoplasty consisted of resection of supra-arytenoidal redundant mucosa, incision of shortened aryepiglottic folds, epiglottopexy or a combination of these. Both removal of supra-arytenoidal redundant mucosa and incision of the aryepiglottic folds were performed with either cold steel instrumentation or (preferably) CO_2_ laser (0.8–1.5 W, superpulse, continuous mode, spot size 200 μm). Epiglottopexy consisted of de-epithelialization of the vallecula and suturing the lingual side of the epiglottis to the base of the tongue. Airway management consisted of either orotracheal intubation whether or not with interrupted apnea, or (supraglottic) high frequency jet ventilation (SHFJV).

Post-operative care depended on severity of preoperative symptoms. It usually consisted of immediate extubation and one night observation with cardiorespiratory monitoring. No preventive antibiotic treatment was administered. Occasionally patients were treated with dexamethasone (0.5–1 mg/kg). If no events occurred during observation patients were discharged the day after surgery.

Standard follow-up was performed 6 weeks after initial endoscopy. The frequency of further controls was depending on the complaints. Patients with no complaints were dismissed from regular follow-up. If no/insufficient improvement or progression of the symptoms occurred after wait-and-see or supraglottoplasty, a second diagnostic endoscopy was performed (median 43 weeks; range 260 weeks). If laryngomalacia was still apparent after wait-and-see, this was called residual disease. If laryngomalacia was still apparent after supraglottoplasty, it was called recurrent disease. During the last control, severity of symptoms was divided into no improvement, partial improvement or complete improvement.

Statistical analysis was performed using SPSS version 20.0. For comparison between groups we used the Mann–Whitney *U* test. For correlations the Spearman test was performed. Statistical significance was set at *p* < 0.05.

## Results

A total of 100 patients met our inclusion criteria, based on the registered diagnosis. After analysis of data 11 patients were excluded. Ten patients were excluded because of misdiagnosis after reviewing photo/video documentation and OR-reports [bilateral vocal cord paralysis (*N* = 5), laryngeal web (*N* = 1), luxated arytenoids (*N* = 1), retrognatia (*N* = 1) and tracheamalacia (*N* = 2)]. One patient was excluded due to lack of data to objectify laryngomalacia. In total, 89 patients remained for statistical analysis.

Table 1 shows characteristics of the study population and Table 2 describes all treatment characteristics. Comorbidity was found in 60 patients (67 %). The most commonly found comorbidity was synchronous airway lesions (SALs) with 36 cases (40 %), showing 54 airway lesions: several patients had more than one synchronous airway lesion. Table 3 shows the different synchronous airway lesions found in this population. Cardiac comorbidity was found in 12 patients (13.5 %), neurologic comorbidity in 8 patients (9.0 %), chromosomal/syndromal disease in 23 patients (25.8 %) and other comorbidities were found in 10 patients (11.2 %).

**Table 1:** Patient characteristics of 89 patients treated for laryngomalacia

Age in days, median (range)	
Presentation of symptoms	60 (0–1691)
Diagnosis with endoscopy	101 (1–1755)
Sex, *n* (%)	
Male	57 (64)
Female	32 (36)
Gestational age in weeks. Mean (SD)	38 + 4 (2 + 5)
Birth weight in grams. Mean (SD)	3168.6 (850)
Presenting symptoms, *n* (%)	
Stridor	64 (72)
Dyspnea	24 (27)
Apnea	17 (19)
Cyanosis	18 (20)
Feeding problems	12 (13)
Failure to thrive	5 (6)
Respiratory distress	11 (12)
Retractions	15 (17)
Other	3 (3)
Severity of Laryngomalacia, *n* (%)	
Mild	36 (40.4)
Moderate	21 (23.6)
Severe	32 (36.0)
Total	89 (100)
Comorbidities, *n* (%)	
Synchronous airway lesions^a^	36 (40.4)
Cardial^b^	12 (13.5)
Neurological^c^	8 (9.0)
Syndrome/genetic disorders^d^	23 (25.8)
Other^e^	10 (11.2)
None	29 (32.6)

^a^See Table 2

^b^Tetralogy of Fallot (*n* = 2), ventricular septum defect (4), transposition of the great vessels (1), bicuspid aorta valves (1), atrioventricular septum defect and AVSD with pulmonary hypertension (2) or with overriding aorta (1)

^c^Epilepsy (*n* = 3), cerebral infarction (1), cerebral cyst (1), Hirschsprung’s disease (1), gastroparesis and autism (1)

^d^Down syndrome (*n* = 5). Pierre Robin (3), CHARGE (3), Door syndrome (2), West syndrome (2), congenital myasthenia syndrome (1), Rubinstein Taybi syndrome (1), Freeman-Sheldon syndrome (1), Goldenhar syndrome (1), Perlman syndrome (1), macrocephaly-capillar malformation syndrome (1), Q7 syndrome and an unknown mitochondrial syndrome (1)

^e^Hydronephrosis (*n* = 2), pylorus hypertrophy (1), nasal teratoma (1), polycystic kidney disease (1), thyroglossal cyst (1), congenital cataract (1), reflux and hernia umbilicalis combined with phimosis (1)

**Table 2:** Treatment characteristics of 89 patients treated for laryngomalacia

Primary treatment (1st endoscopy), *n* (%)	
Wait-and-see	75 (84.3)
Endoscopic surgery	14 (16.5)
Tracheostomy	0 (0.0)
Recurrent/residual disease (2nd endoscopy), *n* (%)	
After wait-and-see	13 (17.3)
After surgery	1 (7.1)
Treatment of recurrent/residual disease, *n* (%)	
Wait-and-see	3 (21.4)
Endoscopic surgery	9 (64.3)
Tracheostomy	2 (14.3)
Final treatment, *n* (%)	
Wait-and-see	65 (73.0)
Endoscopy	22 (24.7)
Tracheostomy	2 (2.2)
Used technique, *n* (%)	
CO_2_ laser	17 (77.3)
Cold steel	3 (13.6)
Epiglottopexy	2 (9.1)
Complications	0

**Table 3:** Distribution of synchronous airway lesions (SALs) in all patients with laryngomalacia and SAL (*n* = 36)

Synchronous airway lesion	*n* (%)
Number of patients	36 (100)
Tracheamalacia	14 (39)
Pharyngomalacia	13 (36)
Bronchomalacia	9 (25)
Tracheal/subglottic stenosis	7 (19)
Micrognathia	7 (19)
Other^a^	4 (11)
Total SALs	54

^a^Laryngeal web (2), bilateral vocal cord paralysis (1) and missing lung lobe (1)

Presence of any comorbidity was related to more severe form of laryngomalacia (*p* = 0.003). However, no distinction could be made between different types of comorbidities [SAL (*p* = 0.266), cardiac (*p* = 0.115), neurologic (*p* = 0.247) or syndromal/genetic (*p* = 0.026)]. The co-moborbidities showed no correlation with performed treatment. Gestational age, sex, birth weight and age at onset showed no correlations with performed treatment, severity of disease or length of disease. Table 4 shows the influence of comorbidity on the median time to complete improvement. Patients with SALs needed a statistically significant longer time for complete improvement (*p* = 0.043). Although all factors showed difference in median time to complete improvement, no other factors were found to have significant influence on the time to complete improvement. Gender, age at onset, gestational age and birth weight was not related to laryngomalacia severity or time to complete improvement. Multivariate analysis could not be performed due to low number of cases. Only one patient of patient treated with supraglottoplasty (7.1 %) had recurrent disease after supraglottoplasty. During primary supraglottoplasty of this patient, redundant mucosa of only one arytenoid cartilage was removed, based on asymmetric type 1 laryngomalacia. Recurrent disease presented with contralateral type 1 laryngomalacia. During revision supraglottoplasty redundant mucosa over the arytenoid cartilage of the contralateral side was removed. In total 65 (73 %) patients were treated with a wait-and-see policy, 22 (24.7 %) with supraglottoplasty and 2 (2.2 %) with tracheostomy. Of the patients undergoing supraglottoplasty 17 (77.3 %) were performed with CO_2_ laser and 3 (13.6 %) with cold steel instruments.

**Table 4:** Median time in weeks to achieve complete improvement related to comorbidities in patients with laryngomalacia, regardless of treatment modality used

	Present (*n*)	Not present (*n*)	*p*
Comorbidities	31.0 (23)	15.0 (7)	0.116
Cardial	51.0 (5)	26.0 (25)	0.373
Neurologic	40.0 (5)	26.0 (25)	0.845
Syndromal	45.0 (6)	25.5 (24)	0.243
SAL	38.5 (18)	14.5 (12)	**0.043**

Bold value indicates statistically significant *p* value (*p* < 0.05)

Table 5 summarizes the follow up data. Follow-up data were available in 62 patients (70 %). Complete improvement was registered in 30 patients (34 % of total group). In total seven patients died during their follow-up. None of them died due to laryngomalacia: all died of other severe comorbidities. Two patients received a tracheostomy. One of them underwent tracheostomy because the laryngomalacia did not improve and supraglottoplasty was not a standard procedure in our institution yet. However, the other tracheostomy was not related to laryngomalacia, but was performed because of severe tracheamalacia. This was the reason that this patient did not undergo previous supraglottoplasty.

**Table 5:** Outcome of performed treatment in complete group of 62 patients with laryngomalacia, depending on final treatment (*n* (%))

	Final treatment			Total
	Supraglottoplasty	Wait-and-see	Tracheotomy	
Deceased^a^	2	5	0	7
No improvement	1 (4,5)	2 (5.3)	1	4
Partial improvement	9 (40.1)	12 (31.6)	0	21
Complete improvement	10 (45.4)	19 (50.0)	1	30
Total	22 (100)	38 (100)	2	62

^a^Causes of death (all causes occurred once): kidney cancer, epilepsy, tracheamalacia, Freeman-Sheldon syndrome, sepsis, unidentified syndrome, unknown (but not suspicious for laryngomalacia)

Table 6 demonstrates the complete improvement in weeks in patients with different treatment and severity of disease. All patients, who underwent supraglottoplasty, achieved complete improvement within 6 weeks, except three patients who achieved complete improvement in 31, 57 and 58 weeks. Supraglottoplasty showed significant reduction of time to achieve complete improvement in the whole population (*p* = 0.026). However, no statistically significant difference was seen among the different severities of laryngomalacia, very likely due to insufficient numbers. Groups, wait-and-see and supraglottoplasty, matched for gender, age at onset, birth weight and gestational age.

**Table 6:** Median time in weeks (*n*) to achieve complete improvement, depending on severity of disease, and depending on treatment applied

Laryngomalacia	Wait-and-see	Supraglottoplasty	*p*
Mild	32 (13)	–	
Moderate	53 (3)	3 (1)	0.180
Severe	10 (3)	6 (9)	0.778
All severities	29 (19)	5 (10)	**0.026**

Bold value indicates statistically significant *p* value (*p* < 0.05)

## Discussion

Laryngomalacia is a benign and self-limiting disease. The present study confirms the additional value of supraglottoplasty in the treatment of laryngomalacia. We have shown for the first time that supraglottoplasty shortens the length of symptomatic disease, compared to wait-and-see policy. This study also shows that comorbidity, especially synchronous airway lesions, is associated with the severity of disease. Comorbidity tends to negatively influence the resolution of symptoms.

Comorbidity was found in two-thirds of our patients (67 %). According to literature comorbidity is present in around 25–47 % of patient with laryngomalacia, disregarding synchronous airway lesions (SALs) [8, 12, 13]. The higher incidence of comorbidity in our practice is very likely due to the fact, that we also counted SALs as comorbidities and also probably because of the higher ratio of complex patients in our academic setting. Although no other studies directly compared comorbidities with time to complete improvement, comorbidity is found to be risk factors for supraglottoplasty failure [12]. Preciado et al. showed in their review that comorbidity is associated with a more than sevenfold increased risk of surgical failure. Furthermore, otherwise healthy patients have significantly lower risk of post supraglottoplasty aspiration [14, 15]. Durvasula et al. related especially neurologic and syndromic comorbidities with a supraglottoplasty failure [16]. We noted relation between the severity of laryngomalacia and occurrence of comorbidity in general. Furthermore, there also seems to be a negative relation between comorbidity and time to achieve complete improvement; however, this was not statistically significant (*p* = 0.116).

The clinical significance of SALs in patients with laryngomalacia is a rather controversial subject. Studies reporting a high incidence of SALs suggest that SALs are related to disease severity and patients with SALs are therefore more likely to require surgery [17, 18]. Our results suggest that the presence of SALs in patients with laryngomalacia is associated with worse long-term outcome but it is not associated to the severity of disease. On the other hand some studies report no clinical significance of SALs with no influence on time to complete improvement [19, 20], and no association between severity and SALs [21]. The most common reported SAL in our cohort is tracheamalacia (39 %). This is in agreement with other studies [7, 22].

Prevalence of cardiac comorbidity ranges between 10 and 17.9 % in patients with laryngomalacia in the literature [2, 8, 23]. Patients with cardiac comorbidities have a higher chance of revision supraglottoplasty or tracheostomy and are associated with increased severity [8]. This could not be confirmed by our data. However, cardiac impairment was of negative influence on time to achieve complete improvement, but this correlation was not statistically significant (*p* = 0.373).

Neurologic impairment is thought to influence laryngomalacia severity and outcome [2, 8, 12, 16, 17, 24, 25]. All these studies related neurologic comorbidity to increased chance of revision supraglottoplasty and tracheostomy. However, we cannot relate neurologic comorbidities to worse outcome in our series, as the patient with revision supraglottoplasty and the two tracheostomy cases did not have any neurologic conditions.

The reported incidence of syndromal and genetic disorders ranges from 8 to 20 % [1]. Down syndrome, CHARGE and Pierre Robin are the most frequently seen disorders in literature and also in our population [8]. However, the significance of the presence of syndromal/genetic disorders has not been well examined. Syndromes associated with retrognathia, like CHARGE and Pierre Robin sequence, are more likely to fail supraglottoplasty [8, 16, 26]. Our data show that syndromal/genetic disorders are related to the severity of disease. Our data show a trend that patients with syndromal/genetic disorders need more time to achieve complete improvement than other patients (*p* = 0.243).

We found only one patient with gastroesophageal reflux disease (GERD), where the reported incidence is between 65 and 100 % [1, 27, 28]. A systematic review of 27 studies showed coexistence between GERD and laryngomalacia. However, due to limited evidence no causal connection could be made [27]. Due to the retrospective nature of the underlying study it is hard to determine whether reflux was not present. However, video and photo documentation showed no convincing signs of GERD. Studies reporting high incidence lack a control group, thus the clinical significance of the presence of GERD has not been well established [27]. Most probably, considering literature data, GER and not GERD was present in our cohort but not documented. The presence of GERD and the clinical significance of it should be further examined.

A noteworthy finding in our study is the low amount of revision supraglottoplasty, tracheostomies and complications. Hoff et al. described a higher rate of revision supraglottoplasty (15.2 vs. 47.8 %) and tracheostomy depending on the presence of comorbidity (0 vs. 39.1 %) [8]. Other studies showed a range of 4.4–34.6 % of patients required either revision supraglottoplasty or tracheostomy after primary supraglottoplasty [12, 29, 30]. Although complications are rare [31], supraglottic stenosis is a known complication of supraglottoplasty with a reported incidence of 3.7 % [12].

Most of our patients (77.3 %) were operated with CO_2_ laser, with superpulse mode, which is comparable with other authors [8, 29]. Superpulse mode allows the tissue to cool down between two impulses, therefore minimizing tissue damage. Rastatter et al. used both CO_2_ laser and cold instruments techniques. Only in the CO_2_ laser supraglottoplasty group three patients needed tracheostomy [30], suggesting CO_2_ laser to be less safe. In contrast, 80 % of patients in the study of Denoyelle et al. [12], with a reported rate of 3.7 % laryngeal stenosis, were operated using cold steel micro-instruments. Other factors, like the application of dexamethasone or supraglottic high frequency jet ventilation (SHFJV) [32], which improves surgical precision by providing good exposure of the operating area, might have also contributed to our low complication rate.

Without doubt, there are some weaknesses of the present study. Missing data were to be expected due to the retrospective nature of this study because of incomplete data registration in the paper and electronic charts. In addition, this study contained insufficient numbers to perform a multivariate analysis. Patients with more severe disease were more likely to undergo supraglottoplasty, resulting in a selection bias in the operated group of patients. Some patients with supraglottoplasty indicated at the second endoscopy were already candidates for surgery at the initial endoscopy. Despite this potential selection bias, patients who underwent supraglottoplasty were found to need significantly shorter time to achieve complete improvement. A prospective, multicenter study could solve these problems; however, uniformity in diagnostics and treatment might be the drawback of such a study.

## Conclusion

To our best knowledge, this is the first study relating the outcome of supraglottoplasty with time. Our results show that supraglottoplasty is beneficial in the treatment of patients with severe laryngomalacia by significantly reducing the time to improvement of the complaints. Furthermore, comorbidity is found to have influence on the duration and severity of laryngomalacia.

## Electronic supplementary material

Below is the link to the electronic supplementary material.

Supplementary material 1 (WMV 528 kb)

Supplementary material 2 (WMV 466 kb)

Supplementary material 3 (WMV 599 kb)
